# Risk Prediction Score for Pediatric Patients with Suspected Ebola Virus Disease

**DOI:** 10.3201/eid2806.212265

**Published:** 2022-06

**Authors:** Alicia E. Genisca, Tzu-Chun Chu, Lawrence Huang, Monique Gainey, Moyinoluwa Adeniji, Eta N. Mbong, Stephen B. Kennedy, Razia Laghari, Fiston Nganga, Rigo F. Muhayangabo, Himanshu Vaishnav, Shiromi M. Perera, Andrés Colubri, Adam C. Levine, Ian C. Michelow

**Affiliations:** Brown Emergency Medicine, Providence, Rhode Island, USA (A.E. Genisca, H. Vaishnav, A.C. Levine);; Alpert Medical School of Brown University, Providence (A.E. Genisca, A.C. Levine, I.C. Michelow);; University of Georgia, Athens, Georgia, USA (T.C. Chu);; Brown University, Providence (L. Huang, M. Adeniji);; Rhode Island Hospital, Providence (M. Gainey);; International Medical Corps, Goma, Democratic Republic of the Congo (E.N. Mbong, R. Laghari, F. Nganga, R.F. Muhayangabo);; Ministry of Health, Monrovia, Liberia (S.B. Kennedy);; International Medical Corps, Washington, DC, USA (S.M. Perera);; University of Massachusetts Chan Medical School, Worcester, Massachusetts, USA (A. Colubri)

**Keywords:** Ebola virus disease, risk prediction score, children, viruses, West Africa

## Abstract

Rapid diagnostic tools for children with Ebola virus disease (EVD) are needed to expedite isolation and treatment. To evaluate a predictive diagnostic tool, we examined retrospective data (2014–2015) from the International Medical Corps Ebola Treatment Centers in West Africa. We incorporated statistically derived candidate predictors into a 7-point Pediatric Ebola Risk Score. Evidence of bleeding or having known or no known Ebola contacts was positively associated with an EVD diagnosis, whereas abdominal pain was negatively associated. Model discrimination using area under the curve (AUC) was 0.87, which outperforms the World Health Organization criteria (AUC 0.56). External validation, performed by using data from International Medical Corps Ebola Treatment Centers in the Democratic Republic of the Congo during 2018–2019, showed an AUC of 0.70. External validation showed that discrimination achieved by using World Health Organization criteria was similar; however, the Pediatric Ebola Risk Score is simpler to use.

Ebola virus disease (EVD) is a potentially fatal infectious disease, easily transmitted through direct contact with infected body fluids. Children exhibit a range of nonspecific clinical signs that mirror common endemic febrile diseases, such as malaria and gastroenteritis. Few children experience hemorrhage, and some are afebrile (*1*). The 2014–2016 West Africa Ebola outbreak was the largest EVD epidemic in history; 28,646 cases were suspected, probable, or confirmed, of which nearly 20% occurred in children <15 years of age, and 11,323 case-patients of all ages died (*2*). EVD quickly became a global public health concern as 7 other countries, including the United States, reported cases (*3*). Since then, there have been several outbreaks in the Democratic Republic of the Congo (DRC), the largest of which occurred during 2018–2020 in the North Kivu, Ituri, and South Kivu Provinces.

Our research and that of others previously showed young children to be especially vulnerable and susceptible to EVD; mortality rates exceeded 55% (*1*,*4*). Consequently, there is a critical need to rapidly diagnose EVD in children so they can be appropriately isolated and begin treatment. During EVD outbreaks, triage protocols are typically based on World Health Organization (WHO) criteria for screening children with suspected EVD. According to WHO criteria, a suspected case-patient is defined as anyone, dead or alive, who has been in contact with someone with a suspected, probable, or confirmed EVD case; has sudden onset of fever combined with >3 other signs/symptoms; has inexplicable bleeding; or suddenly inexplicably died in the context of an EVD outbreak (*5*). Therefore, we adopted age-dependent case definitions: a fever and 1 other sign/symptom for children <5 years of age, 2 other signs/symptoms for children 5–12 years of age, and >3 signs/symptoms for children >12 years of age (*6*). However, nonspecific signs/symptoms in the early stages of disease impede prompt and accurate identification of cases and result in poor discrimination when applying the WHO broad case definitions. In addition, if EVD-negative children are unnecessarily admitted to Ebola treatment centers (ETCs), they require use of scarce resources and are potentially exposed to EVD case-patients. There is a critical knowledge gap in clinical diagnostics for children with EVD; few published studies focus on the epidemiology and diagnosis of pediatric EVD (*4*,*6*). To our knowledge, 1 study has created a diagnostic predictive score for pediatric EVD (*6*), but those results have not been externally validated.

Although great strides in EVD care have been made with the advent of highly effective vaccines and treatments (*7*–*9*), an accurate predictive clinical diagnostic tool can be helpful for clinicians before molecular test results are available. Such a tool would help streamline the triage process, enhancing the ability of clinicians to rapidly identify children at the highest risk for EVD, initiate time-sensitive treatment, and protect EVD-negative children from nosocomial acquisition of EVD.

With this study, we addressed the knowledge gaps associated with management for children with suspected EVD by developing a predictive diagnostic tool. Ethics approval for this study was exempted by the Rhode Island Hospital Institutional Review Board because it is a secondary analysis of deidentified data.

## Materials and Methods

### Data Sources

Our retrospective study used data that had been prospectively collected from children at the International Medical Corps (IMC) ETCs in West Africa (West Africa cohort) and the DRC (DRC cohort). The derivation dataset was built from data collected at 5 IMC ETCs in Sierra Leone and Liberia during September 2014–September 2015. The validation dataset was derived from children who were at the IMC Mangina ETC in the DRC during December 2018–December 2019. For the derivation and the validation datasets, we systematically extracted data from paper clinical records, which were scanned by ETC staff onto the IMC secure server. Research staff then transcribed the information into respective databases and removed all personal identifiers before analysis.

### Data Quality Audit

For the derivation and validation datasets, all data were deidentified before analysis. To ensure minimal errors during data entry, we took the following steps: used data validation settings in Excel documents; used codebooks to ensure that patient data were standardized; had data entry research coordinators conduct additional audits; and discussed data entry concerns with the principal investigator. We used a random sample of charts to assess the quality of data entered from original patient charts into the database for EVD-positive persons. We selected 19 patients for the derivation dataset and 62 patients for the validation dataset and included them in the data quality audit, in which patient charts were reentered into a second database by using scanned files of the original charts (*10*). After reentry was complete, we compared the original data to the reentered database for each respective cohort and recorded each discrepancy as an error. With results from this audit, we concluded that, overall, 99.8% of data were entered correctly in the derivation dataset and 97.3% of the data in the original database were consistent with information from the scans of patient charts for the validation dataset (*10*).

For additional quality assurance, we compared the validation dataset’s more simplified line list database and the EVD-positive database across 145 common variables to check for any inconsistencies. If any fields were flagged, we referenced the paper charts for further clarification and resolved in both databases.

### Inclusion and Exclusion Criteria

For the derivation and the validation datasets, all pediatric patients (<18 years of age) with suspected EVD who were admitted to any of the ETCs were eligible for study inclusion. We excluded from analysis patients for whom all clinical sign/symptom data were missing. We also excluded patients who died within the first 24 hours after admission because a diagnostic tool would probably be less useful for severely ill patients whose death was imminent.

### EVD Triage and Diagnosis

Trained clinical staff screened all patients at the IMC ETCs according to WHO and Médecins Sans Frontières guidelines (*11*,*12*) as well as individual clinicians’ judgment. Patients with a previously confirmed laboratory diagnosis of EVD were directly admitted to the ETC confirmed ward. Otherwise, patients who met the definition of having a suspected case were admitted to the ETC suspected ward, where they had a blood sample drawn for initial EVD testing (Appendix). If the patient’s initial test result was negative, the patient remained in the ETC until a second test ruled out EVD. Patients with a second negative test result were considered EVD negative and discharged. Patients with a positive test result were considered EVD positive and moved to the confirmed ward for further management (E.N. Mbong, unpub. data) (*10*,*13*).

### West Africa: Liberia and Sierra Leone

In Liberia, ETCs received all patients from the surrounding catchment areas. However, in Sierra Leone, multiple agencies operating in the ETC districts and the government-run District Ebola Response Center determined to which ETCs patients should be sent. In both countries, most patients seen at the ETC had >1 signs/symptoms consistent with EVD but no laboratory confirmation. Some may have had EVD confirmed in community or government-managed holding centers before arrival at the ETC (*10*,*13*).

For Liberia, the US Naval Medical Research Center Mobile Laboratory (Frederick, Maryland, USA) conducted the 1-step quantitative Ebola Zaire real-time reverse transcription PCR (RT-PCR) (Taqman) assay for both IMC ETCs. For this assay, they used a QIAamp Viral RNA Mini Kit (https://www.qiagen.com) to extract RNA from blood samples treated with QIAGEN buffer AVL and ethanol. Using the Applied Biosystems StepOnePlus instrument (https://www.thermofisher.com), they tested the extracted RNA for 2 Ebola virus (EBOV) gene targets (Zaire ebolavirus locus and minor groove binding locus). If both targets were detected, a sample was considered positive for EVD. If only 1 target was detected, the sample was considered indeterminate, and the patient was retested (*10*,*13*).

In Sierra Leone, the Public Health England (PHE) laboratories in Port Loko and Bombali districts performed EVD testing for patients admitted to ETCs in those districts, and the Nigeria laboratory in Kambia District provided RT-PCR testing for patients admitted to the Kambia ETCs with support from the European Union Mobile Laboratory Consortium. The PHE and Nigeria laboratories tested only 1 EBOV gene target (Zaire ebolavirus locus). In February 2015, the PHE laboratories switched from using the commercially available Altona real-time RT-PCR to the in-house Trombley assay (*10*,*13*).

### DRC

DRC ETCs received all patients from the surrounding catchment areas, some of whom may have had EVD confirmed by laboratory testing in the community or another test facility before arrival. EVD diagnoses were made by using a Cepheid GeneXpert Ebola RT-PCR blood assay (https://www.cepheid.com) targeting 2 EBOV genes: glycoprotein and nucleoprotein (*14*,*15*). Laboratory testing was conducted by the Institut National de Recherche Biomédicale (Kinshasa, DRC). All cycle threshold values presented in this study are based on RT-PCR. Cycle threshold values >40 were considered negative for all cases.

### Statistical Analyses

We described the demographic and clinical characteristics of the study population according to EVD status by using frequencies with percentages for categorical variables and median values with interquartile ranges (IQRs) for continuous variables. We performed univariate analyses to evaluate associations between candidate predictors and EVD status and reported odds ratios (ORs) with 95% CIs.

**Figure 1:**
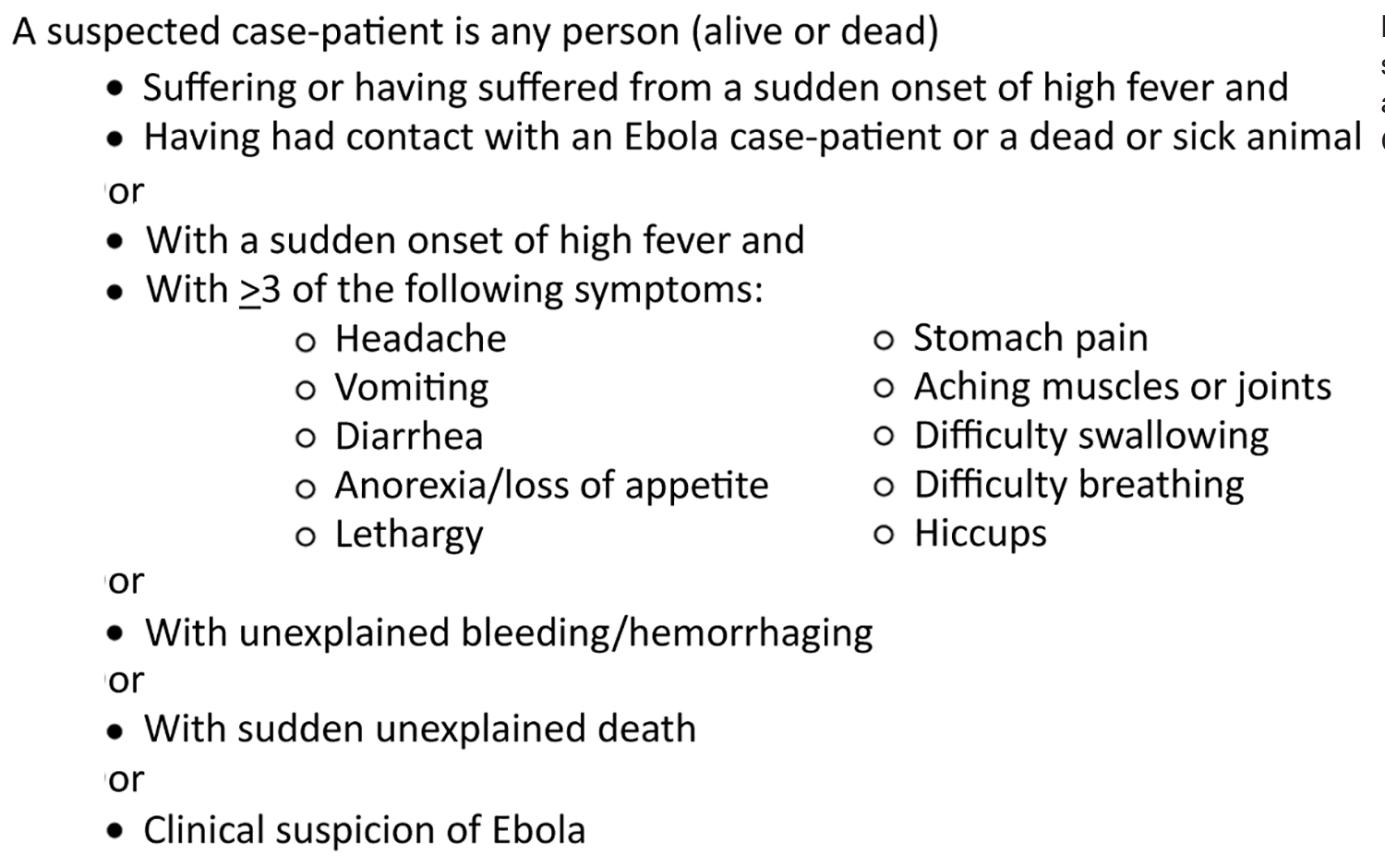
Ebola virus disease suspected case definition according to 2016 World Health Organization guidelines.

The 12 candidate predictors were age, sex, and 10 other epidemiologic and clinical variables based on the current WHO criteria (Figure 1) for identifying suspected Ebola cases (fever, headache, breathlessness, bone or muscle pain, asthenia, abdominal pain, hiccups, unexplained bleeding, gastrointestinal symptoms [vomiting, diarrhea, nausea, anorexia or swallowing problems], and contact with an EVD case-patient [Ebola contact]). Ebola contact was a composite variable consisting of a combination of 11 individual variables associated with potential contact with an EVD case-patient. These variables included contact with a known/suspected EVD case-patient or any sick person in the previous 21 days; contact with the body, body fluids, or potentially contaminated objects or eating utensils; shared living space with an EVD patient/sick person; attendance at a funeral or contact with the infected body at a funeral; travel outside the patient’s home/village; hospitalization or visit with a hospitalized patient; consultation with a traditional healer; or direct contact with animals or raw meat (hunting/touching/eating). To use the complete dataset, we created 3 categories for Ebola contacts: yes, no, or no known.

### Derivation of Clinical Diagnostic Model

We entered 12 candidate predictors into a logistic regression model to predict EVD diagnosis by using a forward stepwise regression algorithm with 10-fold cross-validation as previously described (*16*). We modeled clinical symptom predictors as dichotomous variables and Ebola contacts as 2 indicator variables and used no contact as the reference. We explored models with interactions. Age was fitted as a linear variable and as restricted cubic splines with 3 knots located at the 10th, 50th, and 90th quantiles. We selected the model without restricted cubic splines or interaction terms because that model performed the best.

### Model Performance and Development of a Risk Score

We assessed the discrimination for the derived model and newly created risk score compared with the WHO criteria. Model discrimination was evaluated by using the area under the receiver operating characteristic curve (AUC) and its 95% CIs at consecutive threshold settings of the predicted probability (*17*,*18*). We developed a point-based risk score (Pediatric Ebola Risk Score; PERS) by converting the regression coefficient of each predictor in the final model to an integer (*19*). We then calculated a total score for each patient by adding these weighted risk scores. The performance of the PERS was also evaluated in the same fashion as the original model. Other performance measures of PERS and WHO criteria at each cut point were also estimated for EVD diagnosis, including sensitivity, specificity, positive predictive value (PPV), negative predictive value (NPV), and positive and negative likelihood ratios.

### External Validation and Model Updating

We externally validated our PERS tool with the DRC dataset by using the same inclusion criteria as used for the derivation dataset. We performed bivariate analyses to compare baseline characteristics between the West Africa and DRC cohorts by using χ^2^ tests. To assess the performance of PERS versus WHO criteria in the DRC cohort, we calculated the AUC, sensitivity, specificity, PPV, NPV, and positive and negative likelihood ratios. All analyses were conducted by using R version 4.0.3 (R Foundation for Statistical Computing, https://www.r-project.org) and Stata version 16.0 (StataCorp, https://www.stata.com).

## Results

### Enrollment and Baseline Characteristics

**Figure 2:**
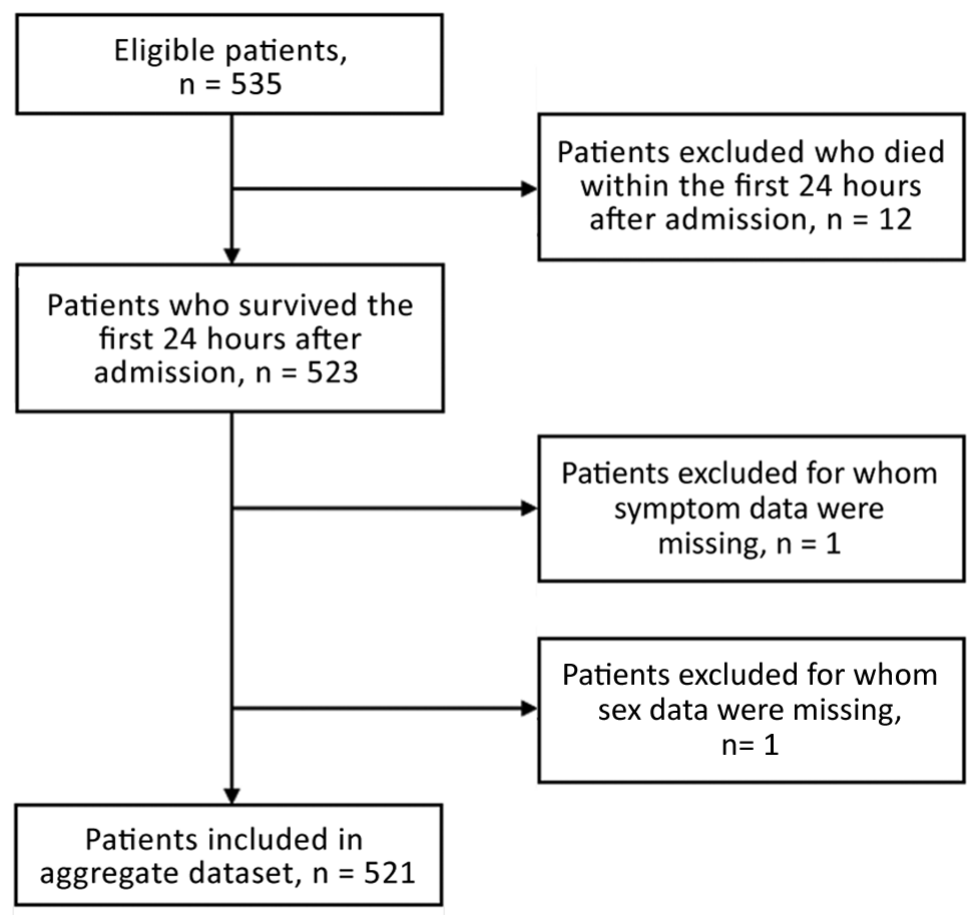
Selection process for West Africa (derivation) dataset during model development for study of risk prediction score for pediatric patients with suspected Ebola virus disease in West Africa.

During September 2014–September 2015, a total of 535 patients <18 years of age at IMC West Africa ETCs with suspected EVD were eligible for inclusion. We excluded from analysis 12 patients who died within the first 24 hours after admission, 1 patient for whom sex classification was missing, and 1 patient for whom all sign/symptom data were missing, leaving 521 patients in the final derivation analysis (Figure 2). Median patient age was 7 (IQR 3–13) years, and 261 (50%) patients were male (Table 1).

**Table 1:** Demographic and clinical characteristics of patients, by EVD status at triage, in West Africa, September 2014 – September 2015*

Characteristics	Total, no (%), n = 521	EVD positive, no. (%), n = 120 (23%)	EVD negative, no. (%), n = 401 (77%)	OR (95% CI)	p value
Sex					
M	261 (50)	53 (44)	208 (52)	0.73 (0.49–1.10)	0.14
F	260 (50)	67 (56)	193 (48)	Referent	
Sign/symptom					
Fever	431 (83)	95 (79)	336 (84)	0.74 (0.44–1.25)	0.24
Headache	268 (51)	54 (45)	214 (53)	0.71 (0.47–1.08)	0.11
Breathlessness	84 (16)	16 (13)	68 (17)	0.75 (0.41–1.33)	0.35
Bone/muscle pain	201 (39)	43 (36)	158 (39)	0.86 (0.56–1.31)	0.48
Asthenia	333 (64)	77 (64)	256 (64)	1.01 (0.67–1.56)	0.95
Abdominal pain	219 (42)	29 (24)	190 (47)	0.35 (0.22–0.56)	**<0.001**
Hiccups	39 (7.5)	5 (4.2)	34 (8.5)	0.47 (0.16–1.13)	0.12
Any bleeding	77 (15)	36 (30)	41 (10)	3.76 (2.26–6.25)	**<0.001**
GI symptoms	355 (68)	73 (61)	282 (70)	0.66 (0.43–1.01)	0.05
Ebola contact					**<0.001**
Yes	218 (42)	104 (87)	114 (28)	31.3 (15.1–76.1)	
No known	56 (11)	9 (7.5)	47 (12)	6.57 (2.33–19.2)	
No	247 (47)	7 (5.8)	240 (60)	Referent	
Malaria					**0.009**
Yes	163 (31)	27 (23)	136 (34)	0.42 (0.24–0.73)	
Missing†	233 (45)	53 (44)	180 (45)	0.63 (0.39–1.02)	
No	125 (24)	40 (33)	85 (21)	Referent	

*Patient median age (interquartile range) = 7 (3–13) y; OR (95% CI) = 1.00 (0.97–1.04); p = 0.80. Boldface indicates statistical significance. EVD, Ebola virus disease; GI, gastrointestinal; OR, odds ratio. †Missing refers to patients who did not have a rapid diagnostic test completed or results not available.

### Derivation of Predictive Diagnostic Model for EVD

Of the 12 candidate predictors included in the bivariate analyses, 3 variables were significantly positively associated with an EVD diagnosis: bleeding (OR 3.76, 95% CI 2.26–6.25), a reported Ebola contact (OR 31.3, 95% CI 15.1–76.1), and no known Ebola contact (OR 6.57, 95% CI 2.33–19.2). Abdominal pain (OR 0.35, 95% CI 0.22–0.56) was negatively associated with an EVD diagnosis (Table 1).

### Risk Score Assessment and Validation

**Figure 3:**
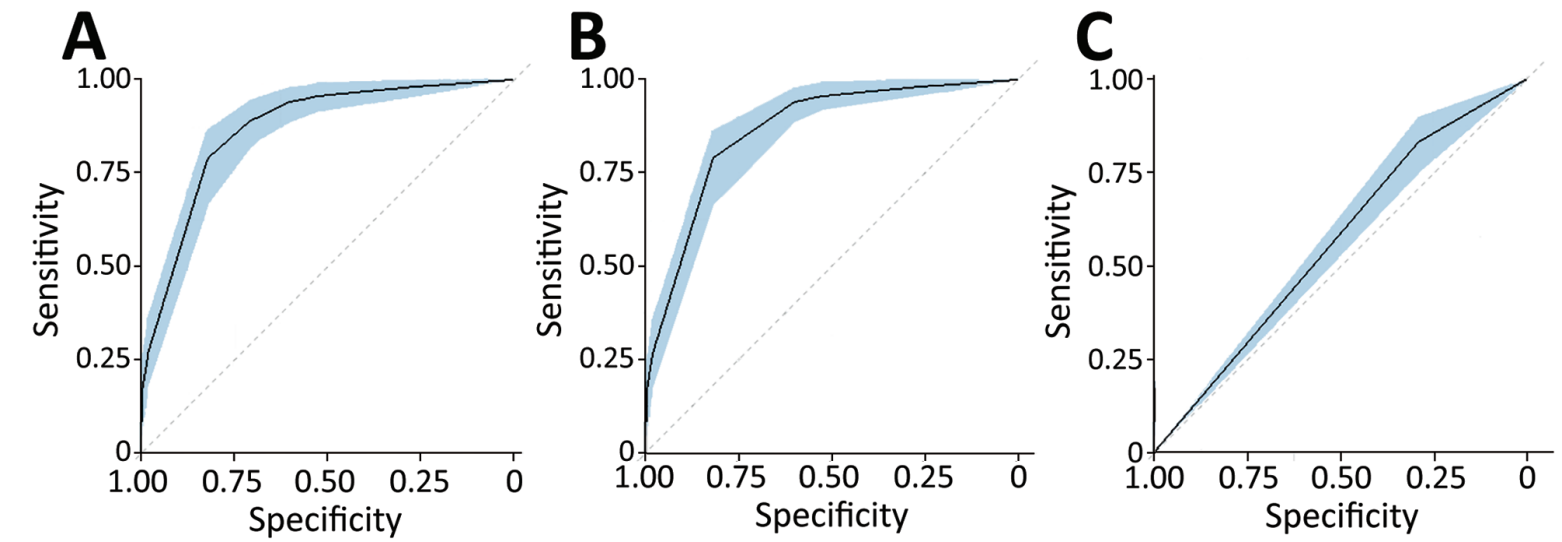
Comparison of strength of discrimination using areas under the curve for study of risk prediction score for pediatric patients with suspected Ebola virus disease in West Africa. A) Ebola diagnostic model; B) Pediatric Ebola Risk Score; C) World Health Organization criteria. The shaded blue regions within each of the panels represent the confidence bands for the areas under the curve.

Forward stepwise regression yielded a final model consisting of 3 covariates: abdominal pain, any bleeding, and Ebola contact without inclusion of interaction terms. The regression coefficients for each variable were converted into integer scores, producing a 7-point scoring system (Table 2). The sensitivity and specificity of the various score cut points for determining EVD status were calculated; higher score cut points were more specific and less sensitive (Table 3). Model discrimination, measured by using the AUC, was 0.87 (95% CI 0.83–0.90) for EVD diagnostic model and point-based risk score (Figure 3). According to the WHO criteria for this dataset, the AUC is 0.56 (95% CI 0.52–0.60).

**Table 2:** Ebola diagnostic model and corresponding point risk score in West Africa, September 2014–September 2015

Variable	Regression coefficient (95% CI)	Odds ratio (95% CI)	Risk score
Ebola contact			
No	Referent	Referent	0
Yes	3.55 (2.78 to 4.49)	34.9 (16.1 to 89.2)	3
No known	1.88 (0.81 to 3.00)	6.56 (2.24 to 20.0)	2
Any bleeding			
No	Referent	Referent	0
Yes	2.02 (1.31 to 2.77)	7.51 (3.70 to 16.0)	2
Abdominal pain			
No	Referent	Referent	0
Yes	−1.19 (−1.80 to −0.63)	0.30 (0.17 to 0.53)	−1

**Table 3:** Performance measures of Pediatric Ebola Risk Score at different cut points and WHO criteria in West Africa cohort, September 2014 – September 2015

Measure	Measure, % (95% CI)					
	Sensitivity	Specificity	PPV	NPV	LR+	LR–
Score						
>0	98.3 (94.1–99.8)	26.2 (21.9–30.8)	28.5 (24.2–33.1)	98.1 (93.4–99.8)	1.33 (1.25–1.42)	0.06 (0.02–0.25)
>1	95.8 (90.5–98.6)	52.4 (47.3–57.3)	37.6 (32.1–43.3)	97.7 (94.7–99.2)	2.01 (1.8–2.24)	0.08 (0.03–0.19)
>2	94.2 (88.4–97.6)	60.1 (55.1–64.9)	41.4 (35.5–47.5)	97.2 (94.3–98.9)	2.36 (2.08–2.68)	0.10 (0.05–0.2)
>3	79.2 (70.8–86.0)	81.8 (77.7–85.4)	56.6 (48.7–64.2)	92.9 (89.7–95.4)	4.35 (3.47–5.46)	0.25 (0.18–0.36)
>4	26.7 (19.0–35.5)	98.0 (96.1–99.1)	80.0 (64.4–90.9)	81.7 (78.0–85.1)	13.4 (6.33–28.2)	0.75 (0.67–0.83)
WHO criteria	83.3 (75.4–89.5)	28.9 (24.5–33.6)	26.0 (21.7–30.7)	85.3 (78.2–90.8)	1.17 (1.06–1.30)	0.58 (0.38–0.88)

*LR+, true positive/false positive likelihood ratio; LR–, false negative/true negative likelihood ratio; NPV, negative predictive value; PPV, positive predictive value; WHO, World Health Organization.

### External Validation

**Figure 4:**
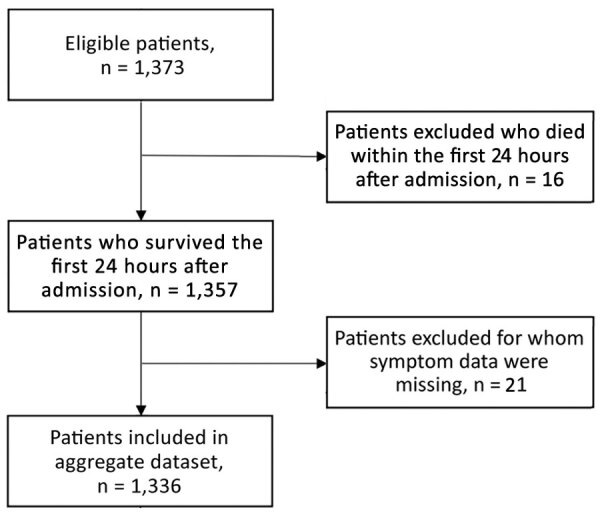
Selection process for Democratic Republic of the Congo (validation) dataset for study of risk prediction score for pediatric patients with suspected Ebola virus disease in West Africa.

**Figure 5:**
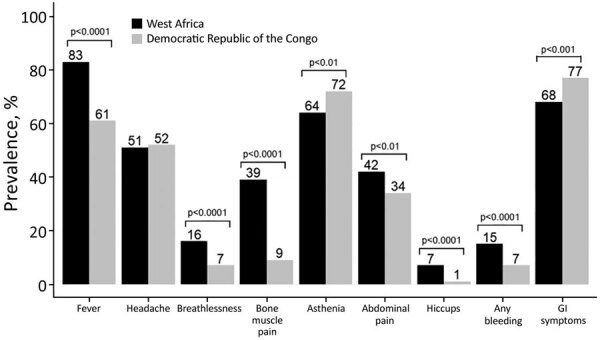
Prevalence of clinical symptoms for pediatric patients with suspected Ebola virus disease in West Africa, September 2014–September 2015, compared with Democratic Republic of the Congo, 2018–2019.

We included 1,336 patients in the final validation dataset after excluding 16 patients who died within the first 24 hours of admission and 21 for whom any sign/symptom data were missing (Figure 4). For the DRC cohort at triage (Table 4), median age of patients in the validation cohort was 7 (IQR 2–11) years and 52% were male, similar to the West Africa cohort. In terms of clinical signs/symptoms for patients in the 2 cohorts (Figure 5), prevalence of fever, breathlessness, and bone/muscle pain was significantly higher among those in the West Africa cohort (p<0.0001), and gastrointestinal signs/symptoms were significantly higher among those in the DRC cohort (p<0.001).

**Table 4:** Demographic and clinical characteristics of patients, by EVD status at triage, in Democratic Republic of the Congo, December 2018–December 2019*

Characteristic†	Overall, no. (%), n = 1,336	EVD positive, no (%), n = 84 (6%)	EVD negative, no. (%), n = 1,252 (94%)	OR (95% CI)	p value
Sex					
M	690 (52)	32 (38)	658 (53)	0.56 (0.35–0.87)	0.01
F	646 (48)	52 (62)	594 (47)	Referent	
Signs/symptoms					
Fever	818 (61)	72 (86)	746 (60)	4.07 (2.27–7.96)	<0.001
Headache	700 (52)	47 (56)	653 (52)	1.17 (0.75–1.83)	0.50
Breathlessness	93 (7.0)	13 (15)	80 (6.4)	2.68 (1.37–4.90)	0.002
Bone/muscle pain	116 (8.7)	16 (19)	100 (8.0)	2.71 (1.47–4.74)	<0.001
Asthenia	960 (72)	62 (74)	898 (72)	1.11 (0.68–1.87)	0.68
Abdominal pain	458 (34)	34 (40)	424 (34)	1.33 (0.84–2.08)	0.22
Hiccups	16 (1.2)	1 (1.2)	15 (1.2)	0.99 (0.05–4.99)	>0.99
Any bleeding	99 (7.4)	21 (25)	78 (6.2)	5.02 (2.86–8.54)	<0.001
GI symptoms	1,026 (77)	84 (100)	942 (75)	55.7 (3.44–900)	0.005
Ebola contact					
Yes	191 (14)	54 (64)	137 (11)	5.40 (3.03–10.1)	<0.001
No known	910 (68)	14 (17)	896 (71)	0.21 (0.10–0.45)	
No	235 (18)	16 (19)	219 (17)	Referent	

*Age, y, mean (interquartile range): overall, 7 (2–11); EVD positive, 5 (1.4–13); EVD negative, 6 (2.5–11); OR 1.00 (95% CI 0.96–1.04); p = 0.96. EVD, Ebola virus disease; GI, gastrointestinal; OR, odds ratio. †Malaria was not reported for this cohort because rapid diagnostic tests for malaria were not conducted for all patients at the EVD treatment centers.

**Figure 6:**
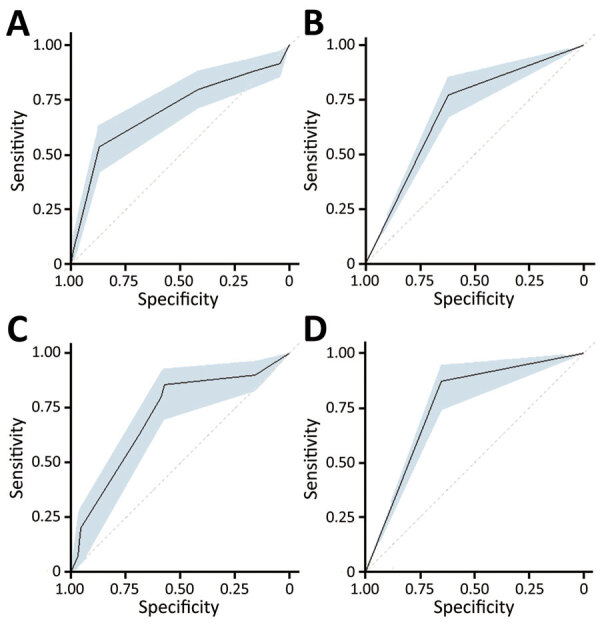
Comparison of strength of discrimination using areas under the curve for Pediatric Ebola Risk Score (PERS) and World Health Organization criteria for study of risk prediction score for pediatric patients with suspected Ebola virus disease in Democratic Republic of the Congo, 2018–2019. A) PERS applied to data including no known Ebola contact (n = 1,336); B) World Health Organization criteria applied to data including no known Ebola contact (n = 1,336); C) PERS applied to data excluding no known Ebola contact (n = 426); and D) World Health Organization criteria applied to data excluding no known Ebola contact (n = 426). The shaded blue regions within each of the panels represent the confidence bands for the areas under the curve.

The performance characteristics of the various score cut points used to determine EVD status by applying the PERS tool to the DRC cohort demonstrated that higher score cut points were more specific and less sensitive, similar to findings for the West Africa cohort (Table 5). Discrimination of the EVD diagnostic model with and without the no known Ebola contact variable was performed by using the DRC cohort. The measured AUC for each model with the no known Ebola contact variable was 0.70 (95% CI 0.63–0.77) and without the variable was 0.71 (95% CI 0.65–0.78). The WHO criteria performed similarly for these datasets (Figure 6).

**Table 5:** Performance measures of Pediatric Ebola Risk Score at different cut points and World Health Organization criteria in Democratic Republic of the Congo cohort, December 2018–December 2019*

Measure	Measure, % (95% CI)						
	Sensitivity	Specificity	PPV	NPV	LR+		LR–
Score							
≥0	91.7 (83.6–96.6)	4.5 (3.4–5.8)	6.1 (4.8–7.5)	88.9 (78.4–95.4)	0.96 (0.90–1.02)	1.86 (0.88–3.96)	
≥1	88.1 (79.2–94.1)	16.3 (14.3–18.5)	6.60 (5.21–8.21)	95.3 (91.6–97.7)	1.05 (0.97–1.14)	0.73 (0.40–1.32)	
≥2	79.8 (69.6–87.7)	41.9 (39.1–44.6)	8.43 (6.59–10.6)	96.9 (95.0–98.2)	1.37 (1.22–1.54)	0.48 (0.31–0.74)	
≥3	53.6 (42.4–64.5)	87.3 (85.3–89.1)	22.1 (16.6–28.4)	96.6 (95.3–97.5)	4.22 (3.30–5.40)	0.53 (0.42–0.67)	
≥4	16.7 (9.42–26.4)	96.4 (95.2–97.4)	23.7 (13.6–36.6)	94.5 (93.1–95.7)	4.64 (2.65–8.10)	0.86 (0.79–0.95)	
WHO criteria	77.4 (67.0–85.8)	62.2 (59.5–64.9)	12.1 (9.45–15.1)	97.6 (96.3–98.6)	2.05 (1.79–2.35)	0.36 (0.24–0.54)	

*Patients with missing Ebola contact information (n = 910) were assigned with a risk score of no known group. LR, likelihood ratio; NPV, negative predictive value; PPV, positive predictive value; WHO, World Health Organization.

## Discussion

In this study, we derived and externally validated a predictive diagnostic model and score for children with EVD. An EVD diagnosis for children was associated with unexplained bleeding, known exposure to an EVD case-patient, or not knowing if the child had come into contact with an EVD case-patient. When converted to a score, the score performed well and showed good discrimination. In addition, the model and score performed similarly or better than the WHO criteria for EVD, the score having the advantage of being simpler and more practical for point-of-care use. Contact with an EVD-positive sick person has been shown to be a strong predictor for EVD diagnosis among adults and children (*6*,*20*). In many studies, bleeding has been shown to be a predictor for poor prognosis (*1*) but is not consistently reported for diagnosis and is usually a late sign in the course of the disease. We found that abdominal pain was negatively associated with an EVD diagnosis.

We externally validated this model and scoring system by using data from the outbreak in the DRC. A PERS >3 had a similar NPV (97%) to the WHO criteria and greater specificity (87%) than the WHO criteria (62%). Therefore, PERS, which is derived from 3 variables compared with 12 variables from the WHO criteria, is a convenient and simple point-of-care tool that can be used by caregivers at the time of triage to rule in EVD and avoid potentially exposing uninfected children to other possible or confirmed EVD case-patients in an ETC. The low PPV of the PERS tool in the DRC probably partly results from a different prevalence of disease (23% in West Africa compared with 6% in DRC). In addition, the percentage of no known Ebola contacts for the DRC cohort (68%) was much larger than that for the West Africa cohort (11%). This finding was a strong diagnostic predictor in the derivation cohort, for which disease prevalence was higher, but it may not have had the same effect in the smaller validation cohort, for which prevalence was lower.

A study limitation is missing epidemiologic and clinical sign/symptom data, which are challenging to collect during an emergency situation, although our data entry error rate was low (after conducting a data quality audit, 99.8% of the West Africa data and 97.3% of DRC re-entry data matched that on the scanned patient charts for patients selected for the data audit) (*10*). In addition, we evaluated only those children who were at the ETCs and met the WHO criteria of having a suspected case. Our findings are not necessarily generalizable to symptomatic children outside this setting.

In summary, using the PERS diagnostic model, we found that Ebola contact status and bleeding were positive predictors of EVD diagnosis, whereas abdominal pain was a negative predictor. The model performed better than the WHO criteria with the West Africa cohort and similarly to WHO criteria with the DRC cohort, yet the PERS model is simpler to use because it requires clinicians to collect only 3 variables rather than 12. Furthermore, using the parsimonious PERS will enable clinicians to promptly triage children with suspected EVD, assign them to cohorts according to their calculated risk for infection, and initiate medical care while awaiting the results of definitive molecular tests. This approach could substantially improve the immediate care of children with suspected EVD and favorably affect their outcomes.

Appendix Additional information for determining risk prediction score for pediatric patients with suspected Ebola virus disease.
