# Supplementary material for: Risk Prediction Score for Pediatric Patients with Suspected Ebola Virus Disease
Source: Emerg Infect Dis. 2022 Jun;28(6):1189–97. doi: 10.3201/eid2806.212265 (PMC9155869; doi:10.3201/eid2806.212265)
Supplement: Appendix — Additional information for determining risk prediction score for pediatric patients with suspected Ebola virus disease. [file 21-2265-Techapp-s1.pdf]

DOI: https://doi.org/10.3201/eid2806.212265

Risk Prediction Score for Pediatric Patients with Suspected Ebola Virus Disease

Appendix

A **suspect case** is any person (alive or dead):

- Suffering or having suffered from a sudden onset of high fever **AND**
- Having had contact with an Ebola case or a dead or sick animal

OR

- With a sudden onset of high fever AND
- With at least three of the following symptoms:
    - Headache
    - Vomiting
    - Diarrhea
    - Anorexia/loss of appetite
    - Lethargy
    - Stomach pain
    - Aching muscles or joints
    - Difficulty swallowing
    - Difficulty breathing
    - Hiccups

OR

- With unexplained bleeding/hemorrhaging

OR

- With sudden unexplained death

OR

- Clinical suspicion of Ebola

Appendix Figure. Ebola virus disease suspected case definition according to 2016 World Health Organization guidelines.
